# Chasing Ebola through the Endosomal Labyrinth

**DOI:** 10.1128/mBio.00346-16

**Published:** 2016-03-22

**Authors:** M. Javad Aman

**Affiliations:** Integrated BioTherapeutics, Inc., Gaithersburg, Maryland, USA

## Abstract

During virus entry, the surface glycoprotein of Ebola virus (EBOV) undergoes a complex set of transformations within the endosomal network. Tools to study EBOV entry have been limited to static immunofluorescence or biochemical and functional analysis. In a recent article in *mBio*, Spence et al. reported a novel, live-cell-imaging method that tracks this transformational journey of EBOV in real time [J. S. Spence, T. B. Krause, E. Mittler, R. K. Jangra, and K. Chandran, mBio 7(1):e01857-15, 2016, http://dx.doi.org/10.1128/mBio.01857-15]. The assay validates known mechanisms of EBOV entry and sheds light on some novel intricacies. Direct evidence supports the hypothesis that fusion is a rare event that starts in maturing early endosomes, is completed in late endosomes, and occurs entirely in Niemann-Pick C1 (NPC1)-positive (NPC1^+^) compartments. The study demonstrated that lipid mixing and productive fusion are temporally decoupled, with different energetic barriers and a protease-dependent step between the two events. Analysis of the mechanism of action of an important class of EBOV neutralizing antibodies, such as KZ52 and ZMapp, provides direct evidence that these antibodies act by inhibiting the membrane fusion.

## COMMENTARY

As obligate intracellular parasites, viruses have to penetrate living cells in order to replicate. While viruses generally have one or more cell surface receptors or attachment factors, only a few virus types can enter the cells through direct fusion with the plasma membrane. Throughout evolution, most viruses have developed elegant strategies to hijack the endosomal network, a maze of tubular and vesicular structures in eukaryotic cells tasked with cellular trafficking, to penetrate the cell and deliver their genome. Endosomes pick up cargo at the plasma membrane and transport it through the cell with the goal of delivering it to the cytoplasm or to other organelles, routing it to the lysosomal graveyard, or recycling back to the plasma membrane. To do this, endosomes undergo a maturation process that is accompanied by morphological and physiochemical transformations, including acidification and acquisition of various functional molecules. Ebola virus (EBOV) utilizes this dynamic endosomal environment to regulate a complex set of transformations of its own envelope glycoprotein that are necessary for fusion of viral and endosomal membranes and delivery of the viral genome into host cells.

To date, studies of the EBOV entry process have been limited to static immunofluorescence imaging of virus particles in “bulk” or biochemical and functional analysis. However, in a recent article in *mBio*, Spence et al. (1) reported a live-cell imaging assay that can track, in real time, this transformational journey of EBOV from the cell surface through the endosomal network and that can directly detect the membrane fusion step in entry. That report, along with a similar assay published recently by Simmons et al. (2), could lead to a deeper understanding of the entry mechanisms of filoviruses and could ultimately help efforts to devise better treatment strategies against these deadly viruses.

**FIG 1:**
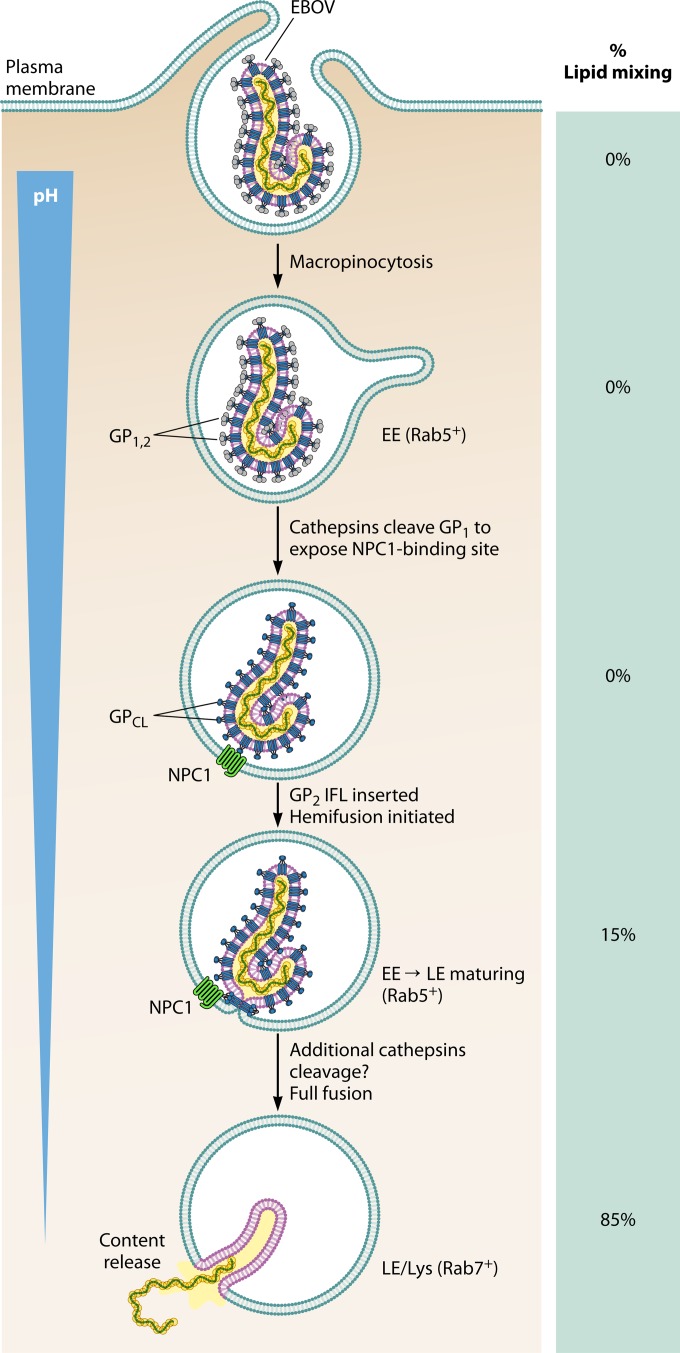
Stages of Ebola virus productive entry into the cells. EE, early endosome; LE, late endosome.

The trimeric glycoprotein (GP) spikes, consisting of the receptor-binding subunit GP1 and the fusion subunit GP2, mediate filovirus entry into host cells. The entry process (Fig. 1) begins with incompletely understood interactions of GP with cell surface attachment factors that deliver virus particles into endosomes via macropinocytosis. Within endosomes, GP undergoes a series of transformations, including proteolytic cleavage and acid-dependent conformational changes, to overcome the high energetic barrier of fusion. Proteolysis of GP in the acidic environment of endosomes by resident cellular enzymes called cysteine cathepsins removes a large portion of the GP1 subunit to unmask the previously buried receptor-binding site (RBS), leaving a trimer of a 19-kDa protein consisting of the entire GP2 and the core of GP1, with the RBS now prominently exposed (3). This cleaved GP (GP_CL_) can now interact with its endosomal receptor, Niemann-Pick C1 (NPC1) (3, 4). The GP_CL_-NPC1 interaction positions the fusion domain to interact with the endosomal membrane and trigger viral membrane fusion.

An enigmatic feature of filovirus entry mechanism is the identity of the “fusion trigger”—the host stimulus that induces the structural rearrangements in GP2 that lead to viral membrane fusion. While GP_CL_-NPC1 interaction is a prerequisite for membrane fusion, it may not be sufficient. Structural analysis has demonstrated that the internal fusion loop (IFL) of EBOV GP undergoes major conformational changes when exposed to acidic pH and the lipid bilayer and that this conformational change may contribute to initiation of fusion (5). In addition to low pH, other factors such as cathepsins may be required for GP triggering, as suggested by the observation that fusion of pseudotype viruses bearing GP_CL_ is inhibited by cathepsin inhibitor E-64 (6, 7). The trigger unwinds the GP2 helical structure from around the GP1 and positions the IFL on the top of the trimer next to the endosomal membrane. The IFL then penetrates the endosomal membrane, and the collapse of this prehairpin intermediate pulls together the virus and endosomal membrane, leading to hemifusion followed by formation of a fusion pore and postfusion six-helix bundle structure (8). The virus then delivers its content through this pore into the host cytoplasm.

To directly visualize the filovirus membrane fusion process, Spence et al. utilized the lipophilic dye DiD to label the envelope of recombinant vesicular stomatitis virus (rVSV) pseudotyped with EBOV GP in various formats. The fluorescent particles were traced as they traversed the endosomal network, and lipid mixing between viral and endosomal membranes—an initial step in the fusion process—was visualized as a rapid increase in DiD fluorescence (“dequenching”) resulting from lateral diffusion of the dye. This detection system was complemented with genetically expressed tags for visualization of various endosomal markers such as Rab5, Rab7, and NPC1 for real-time colocalization analysis. Major strengths of the strategy employed by Spence et al. were that labeling was performed on purified rVSV particles rather than on crude supernatants and that the labeled particles were purified away from unincorporated dye. This dual purification afforded a population of viral particles with uniformly high levels of DiD that yielded a strong increase in fluorescence upon lipid mixing. This feature was critical for detecting the initiation of fusion by a gain-of-function readout rather than by loss of fluorescence.

Studies performed with this live-cell assay have confirmed almost every aspect of EBOV entry previously defined with static, population-based immunofluorescence and biochemical analysis and, at the same time, shed new light on aspects of entry that were previously unknown or underappreciated. The assay confirms that EBOV entry occurs in acidified, NPC1-positive endosomes. By collecting data from a large number of particles, the authors were able to define the half-life (*t*_1/2_) of EBOV entry. Furthermore, the paper showed that the entry process is highly inefficient, as only a small fraction of particles entering the cells actually go on to initiate fusion. This is consistent with reports of high ratios of particles to plaque-forming units or genome copy numbers for filoviruses (9, 10). Such quantitative analysis would have been very difficult, if not impossible, using bulk endocytosis. Moreover, the findings suggest that microscopy-based approaches that fail to directly measure filovirus membrane fusion may lead to misleading conclusions, since most viral particles internalized into endosomes do not initiate productive infection.

Colocalization studies using fluorescently labeled endosomal markers Rab5, Rab7, and NPC1 demonstrated that all virions colocalized with NPC1 at the time of dequenching. This is consistent with two recent reports (2, 11) but is in disagreement with another study suggesting that membrane fusion occurs in endosomes lacking NPC1 but expressing the two-pore calcium channel (TPC) (12). However, in this live-cell assay, the TPC inhibitor tetrandrine did inhibit lipid mixing, consistent with a role for TPC in EBOV entry (12). The study also showed that viral lipid mixing occurs primarily in Rab7-positive (Rab7^+^) late endosomes, consistent with previous reports (2, 11), but that, unexpectedly, about 15% of lipid mixing events occurred in Rab5^+^ endosomes. These Rab5^+^ compartments likely represent maturing vesicles transitioning from early to late endosomes.

An interesting feature of this assay is its ability to uncouple lipid mixing from complete membrane fusion. As mentioned above, the specific cathepsin inhibitor E-64 blocks the entry by rVSV bearing GP_CL_. Interestingly, the live-cell imaging performed by Spence et al. shows that E-64 has no impact on lipid mixing, while it completely blocks productive fusion. These data suggest that there may be an additional protease-dependent step between initial lipid mixing and productive fusion leading to content release. Future studies should reveal the specific target of this proteolytic step and how it relates to the mechanism of fusion.

This assay could be very useful for studying the mechanism of action of entry inhibitors and neutralizing antibodies. The best-characterized neutralizing epitope of EBOV GP is a conformational epitope at the base of the trimeric GP consisting of residues from GP2 and N terminus of GP1. Antibodies such as KZ52 (13), as well as two of the components of the ZMapp therapeutic antibody cocktail (14), bind to this epitope and are believed to block fusion by locking GP in the prefusion conformation (8). For the first time, Spence et al. provided direct evidence in live cells that these antibodies indeed fully block lipid mixing, a precursor for productive fusion. This is consistent with the hypothesis that these antibodies lock the prefusion conformation by bridging GP1 and GP2. Their binding most likely removes the flexibility of the fusion loop so that it cannot undergo the conformational changes needed for insertion into the membrane. Interestingly, a much higher concentration of these neutralizing antibodies was needed for blocking lipid mixing than for virus neutralization, suggesting that productive fusion and delivery of genome face a higher energetic barrier than lipid mixing, possibly because the process requires a higher number of GP spikes to be engaged with the endosomal membrane. Demonstrating this level of mechanistic detail would be nearly impossible without a real-time assay for membrane fusion. In the future, it would be interesting to use this assay to examine how antibodies targeting other types of epitopes such as RBS or glycan cap impact the entry process.

Recently, Simmons et al. also published a similar live imaging assay that is based on pseudotyped retroviral particles labeled with DiD (2). Their work also recapitulated several features of the filovirus entry mechanism, such as dependence on acidified endosomes and cysteine cathepsins, and clearly demonstrated that viral membrane fusion occurs in NPC1^+^ late endosomes/lysosomes (2). However, the level of specific DiD labeling of viral particles in that assay was too low for fluorescence dequenching (i.e., lipid mixing) to be reproducibly monitored. On the other hand, the retrovirus-based particles generated by Simmons et al. included a Gag-mKO fusion protein that is released into the cytoplasm upon productive entry. The ability to show content release is a major strength of this assay, while the ability to show dequenching is a key advantage of the method of Spence et al. It would be highly desirable to combine these two features into one assay as it would allow detailed, real-time study of the decoupled processes of lipid mixing and productive fusion.

Nearly all mechanistic studies on EBOV entry have been performed using pseudotyped viruses. While this system has been very helpful in defining the mechanism of EBOV entry, given the morphological differences between the pseudotypes and the authentic filamentous virus, it is important to replicate these findings using the authentic viruses under conditions of biosafety level 4 (BSL-4) containment. These live-cell-imaging assays are well suited for this purpose as the envelopes can be readily loaded with DiD, and several BSL-4 facilities are equipped with sophisticated imaging systems. Content delivery could be monitored by using recombinant ebolaviruses expressing nucleocapsid proteins with a fluorescent tag. Use of the recently described ΔVP30 EBOV (15) could further simplify such assays by allowing them to be performed under BSL-3 conditions.

Driven by higher frequency of outbreaks with (Zaire) EBOV, nearly all the work on filovirus entry has been focused on this particular virus. It is important to remember that the biology of other filoviruses cannot be simply inferred from that of EBOV. There is considerable sequence divergence between the glycoproteins of EBOV, Sudan virus (SUDV), and Bundibugyo virus (BDBV) and even more between those of ebolaviruses and Marburg viruses. Therefore, it cannot be assumed that the mechanisms of entry for all these viruses are identical. It is hoped that these excellent imaging assays will be also used to shed light on the mechanism of entry of other filoviruses.
